# Dominant inhibition of Fas ligand-mediated apoptosis due to a heterozygous mutation associated with autoimmune lymphoproliferative syndrome (ALPS) Type Ib

**DOI:** 10.1186/1471-2350-8-41

**Published:** 2007-07-02

**Authors:** Lilia L Bi, George Pan, T Prescott Atkinson, Lixin Zheng, Janet K Dale, Christopher Makris, Vishnu Reddy, Jay M McDonald, Richard M Siegel, Jennifer M Puck, Michael J Lenardo, Stephen E Straus

**Affiliations:** 1 Center for Biologics Evaluation and Research, FDA, Rockville, Maryland, USA; 2 Laboratory of Clinical Infectious Diseases, NIAID, NIH, Bethesda, MD 20892, USA; 3 Laboratory of Immunology, NIAID, NIH, Bethesda, MD 20892, USA; 4 Department of Pediatrics, University of California, San Francisco, USA; 5 Autoimmunity Branch, NIAMS, NIH, Bethesda, MD 20892, USA; 6 Department of Pathology, University of Alabama at Birmingham, Birmingham, Alabama, USA; 7 Department of Pediatrics, University of Alabama at Birmingham, Birmingham, Alabama, USA; 8 The Birmingham Veteran's Administration Medical Center, Birmingham, Alabama, USA

## Abstract

**Background:**

Autoimmune lymphoproliferative syndrome (ALPS) is a disorder of lymphocyte homeostasis and immunological tolerance due primarily to genetic defects in Fas (CD95/APO-1; *TNFRSF6*), a cell surface receptor that regulates apoptosis and its signaling apparatus.

**Methods:**

Fas ligand gene mutations from ALPS patients were identified through cDNA and genomic DNA sequencing. Molecular and biochemical assessment of these mutant Fas ligand proteins were carried out by expressing the mutant FasL cDNA in mammalian cells and analysis its effects on Fas-mediated programmed cell death.

**Results:**

We found an ALPS patient that harbored a heterozygous A530G mutation in the FasL gene that replaced Arg with Gly at position 156 in the protein's extracellular Fas-binding region. This produced a dominant-interfering FasL protein that bound to the wild-type FasL protein and prevented it from effectively inducing apoptosis.

**Conclusion:**

Our data explain how a naturally occurring heterozygous human FasL mutation can dominantly interfere with normal FasL apoptotic function and lead to an ALPS phenotype, designated Type Ib.

## Background

ALPS is a heritable disorder of lymphocyte homeostasis due to defects in apoptosis typically involving mutations in genes mediating the Fas/CD95/APO-1 pathway of programmed cell death. Patients with ALPS have chronic, nonmalignant lymphadenopathy and splenomegaly of childhood onset and an increased risk of B-cell lymphomas, autoimmune complications, especially autoimmune cytopenias, increased numbers of normally rare α/βTCR CD3^+^CD4^-^CD8^- ^or "double negative T cells" (DNTs), and defective lymphocyte apoptosis in vitro [1]. Most ALPS patients have genetic abnormalities in the Fas pathway. Fas is a cell surface receptor in the tumor necrosis factor receptor suuper-family and upon stimulation it activates a caspase-mediated apoptosis pathway that controls the homeostasis of mature lymphocytes. ALPS is subdivided into: 1.) Type Ia, ALPS with mutant Fas; 2.) Type Ia, ALPS with somatic mutant Fas in the normally rare population of DNTs; 3.) Type Ib, ALPS with mutant Fas ligand; 4.) Type II, ALPS with mutant caspases; 5.) Type III, ALPS without any defined genetic cause yet. 6.) Type IV, ALPS patients with mutant N-Ras [2]. Majority of ALPS patients are Type Ia and have heterozygous mutations of Fas gene (*TNFRSF6*, tumor necrosis factor receptor gene super-family member. Interestingly, a somatic N-Ras mutation in hemopoietic cells has been found to cause ALPS like syndrome [2]. Homozygous mutations in Fas in both mice and humans are associated with a more severe phenotype than is seen with heterozygous mutations [3-5]. In particular, MRL/*lpr*-/- mice that are homozygous for Fas mutations develop an ALPS-like disease.

The human gene encoding the ligand for Fas (FasL; *TNFSF6*) is located on chromosome 1q23 and spans about 8 kb, including 4 exons that are translated into a 281 amino acid, or approximately 40 kD type II membrane protein. The human and mouse FasL proteins have 77% amino acid identity [6]. Since mice homozygous at the *gld *(generalized lymphoproliferative disease) allele of *TNFSF6 *develop an ALPS-like phenotype [7], FasL defects in humans were predicted to have a similar clinical phenotype.

As in mice, dysfunctional molecules other than Fas that participate in the same signaling pathway, can produce ALPS in humans. Recently, some cases of ALPS, termed Type II and ALPS-like disease have been shown to be due to mutations in caspases 10 and 8, respectively; these are downstream signaling molecules in the Fas pathway [8-13].

The great majority of Fas mutations result in dominant-negative inhibition of apoptosis, due to the random assembly of mutant and normal Fas protein chains at the ligand-independent preassociation stage of Fas trimer formation [14,35]. Penetrance is higher in families with mutations that severely disrupt the Fas death domain [15]. There is striking variability in the degree of severity to which family members bearing the same Fas defective allele express features of ALPS, a variability that is likely explained by modifier genes. For example, our recent studies associate the presence of HLA B44 as protective for clinical ALPS [16]. An additional association study showed caspase-10 polymorphisms might be protective factors in ALPS Type Ia patients [9]. Similar variability is also noted when the murine null allele for Fas, *lpr*, is bred into different strains of mice [17]. The lymphoproliferative features of human ALPS appear to diminish in adulthood [1].

Given that mice harboring homozygous FasL mutations develop chronic lymphadenopathy and autoimmune disease, this led us to look for and ultimately identify an ALPS Type Ib patient, among others, as part of an extended study of patients with features of ALPS to characterize the disorder, its complications, management and causes. One case of a patient with systemic lupus erythematosus (SLE) in an adult has been reported in association with a heterozygous 84 bp in-frame deletion in *TNFSF6 *[18]. That individual was a 64-year-old African-American male with generalized adenopathy but not splenomegaly, malar rash, arthritis, serositis, renal disease, leukopenia, and laboratory features of SLE including positive anti-nuclear and anti-DNA antibodies. Thus, he did not appear typical of ALPS, nor did he meet all ALPS case criteria based on available information. More recently an adult patient with a homozygous allele encoding a FasL protein with a deleterious amino acid substitution in the intracellular portion has been described [19]. She exhibited defective apoptosis and other hallmarks of ALPS. The molecular mechanism underlying the immunopathology appeared to be the absence of any normally functional FasL protein. A number of studies have also reported FasL gene polymorphisms associated with autoimmune conditions such as systemic lupus erythematosus in specific human populations, but others have failed to find such associations in patients with Hashimoto's thyroiditis, Graves' disease, Type I diabetes mellitus, and Sjogren's syndrome [20-25].

In this report we describe a new case of ALPS Type Ib due to a gene mutation that causes a functional alteration of the FasL protein. We found that the mutant protein associates with wild-type FasL and by so doing, dominantly interferes with apoptosis induction. These data strengthen the dominant interference model by which heterozygous FasL gene mutations are capable of causing disease due to impaired apoptosis.

## Methods

### Patient studies

Over a period of 13 years, more than 700 subjects including patients with clinical and laboratory features of ALPS and their relatives have been evaluated by our group at the National Institutes of Health. All subjects were enrolled in institutional review board approved research protocols after written informed consent was obtained. Of these 700 subjects, 240 cases of ALPS were identified: 148 patients (62% of ALPS cases) among 81 families had Fas germline mutations; 4 patients (2%) were positive for somatic mutant Fas in the rare population of DNTs; 2 patients (< 1%), one of whom fulfilled ALPS criteria, had mutant Fas ligand; 7 patients (3%) were positive for mutant caspases; 1 patient (< 1%) was positive for a somatic N-Ras mutation; and the associated apoptosis mutation has yet to be identified in 79 patients (33%) among 76 families.

### Isolation of peripheral blood lymphocytes (PBLs)

Peripheral blood was collected in anticoagulant, and PBLs were isolated by Ficoll-Hypaque density gradient centrifugation. The isolated PBLs were cultured in RPMI 1640 supplemented with 10% fetal bovine serum, 2 mM L-glutamine, 100 μg/ml streptomycin and 100 U/ml penicillin (complete medium) at 37°C in a humidified atmosphere with 5% CO_2_. T cell-enriched populations from PBLs were obtained by stimulation with phytohemagglutinin (PHA, 5 μg/ml, Pharmacia Biotech) in the media for two days and culturing in complete medium for two days followed by culture in medium containing IL-2 (100 U/ml) for a minimum of an additional 2 days until they were used.

### Fas Ab-induced apoptosis

On the day preceding the apoptosis determination experiment, the T cells were fed with fresh IL-2-containing medium. For each sample, 100 μl of cells were plated in triplicate in 96-well plates at a density of 2 × 10^6 ^cells per well. Apoptosis was induced by the addition of 100 μl media containing 1 μg/ml anti-Fas antibody (APO-1-3, Kamiya Biomedical) with 1 μg/ml protein A (Sigma). No antibody was added to control wells. The cells were incubated at 37°C for 24 hours and then assayed for quantification of apoptosis, as reported previously [26]. Briefly, cells were harvested on ice and propidium iodide (final concentration 3.3 μg/ml) was added. Fixed time flow cytometry was performed for 15 minutes to count live cells using a FACScan flow cytometer (Becton-Dickinson). Apoptosis was calculated as:

% cell death = (1 - live cells after antibody treatment/live cells in control tubes) × 100.

### Genomic sequence analysis

Genomic DNA was prepared from activated lymphocytes using standard methods [27]. All four FasL exons and intron/exon boundaries were amplified from genomic DNA with the following PCR primers: Exon 1 fwd 5'-TTG CCT CCT CTT GAG CAG TCA -3'; Exon 1 rev 5'-CAC TTT GCA AGC CAG GCA GT-3'; Exon 2 fwd 5'-GCA GAA CTT CTG AGG TAT TTG GAT TC-3'; Exon 2 rev 5'-CAT TAA CAT AGT TCT GTG CTG AGG ATC-3'; Exon 3 fwd 5'-TAT GTT AGA CTG TTG CCA TTT ACG G-3'; Exon 3 rev 5'-AAG CTT TCC CAA ATC TCA CCT GTA-3'; Exon 4 fwd 5'-AGC TGT CAT TCT GGG TGA AAC ATT-3'; Exon 4 rev 5'-GAC ATT TTG AAC CCT GTG GTC TC-3'. PCR was performed with "Ready To Go" PCR beads (Amersham Pharmacia) as instructed by the manufacturer. After heating for five minutes at 95°C, 30 reaction cycles consisting of 30 sec at 95°C, 30 sec at 60°C and 30 sec at 72°C were performed in a 9700 GeneAmp PCR System (PE Applied Biosystem). The PCR products were loaded on an agarose gel, and the amplicons were excised and purified using purification kits (QIAGEN). The sequences were analyzed with the same primers used for PCR with a dRhodamine Terminator cycle sequencing kit on an ABI 377 DNA sequencer (PE Applied Biosystems). Sequence analyses of the genes encoding Fas and caspase-10 were performed according to methods described in Fisher et al [4] and Wang et al [8].

### Construction of tag-labeled FasL expression vectors (wild type, Pt 86, Pt 55C, and h*gld*)

Complete cDNA of the wild-type human FasL in pBlueScript [6] was used as a template in PCR and cloning reactions. Constructs were generated to express FasL with FLAG or HA tags by inserting 9 amino acids after the cleavage site between amino acids 138 and 139. Five PCR primers were used to amplify the complete FasL cDNA with the tags as two separate fragments. Fragment Ia for FLAG tag was generated by using primer 1 (5'-GGA ATT CCT CAC CAG CTG CCA TGC AGC-3') and primer 2 (5'-TCC CCG CGG CTT GTC ATC GTC GTC CTT GTA GTC GAT ATC GTG GCC TAT TTG CTT CTC C-3'). Fragment Ib for an HA tag was generated by using primer 1 paired with primer 3 (5'-TCC CCG CGG AGC GTA GTC TGG GAC GTC GTA TGG GTA GAT ATC GTG GCC TAT TTG CTT CTC C-3'). Fragment II was generated by using primer 4 (5'-TCC CCG CGG CCC AGT CCA CCC CCT GAA A-3') paired with Primer 5 (5'-GCT CTA GAT TAG AGC TTA TAT AAG CCG-3'). Fragment Ia or Ib was digested with EcoRI/SacII and fragment II was digested with SacII/XbaI. Then fragment Ia or Ib was ligated with fragment II and inserted into the EcoRI/XbaI sites of the pCI-neo expression vector (Promega). The FasL mutations identified in Pt 86 and Pt 55C and the corresponding mouse *gld *mutation were introduced into the FLAG-tagged normal human FasL expression (the *gld *change introduced into the human gene is called *hgld*) construct by site-directed mutagenesis (Stratagene). All of the constructs were verified by sequencing.

### Expression of wild-type or mutant FasL in transfected cells

Human embryonic kidney tumor 293T cells (5 × 10^5^) cultured in DMEM containing 10% FCS and antibiotics were plated onto a 6 well plate and incubated overnight. The cells were then transfected with 0.2~1 μg of plasmid DNA expressing wild type or mutant FasL using the Fugene™ 6-transfection reagent (Roche). To determine whether the mutant FasL molecules could associate physically with wild type (WT) FasL, the HA-tagged WT-FasL and the FLAG-tagged Mut-FasL constructs were co-transfected into 293T cells. The transfection efficiency was above 50% for Jurkat cells and more than 70% for 293T cells as assessed through surface staining of live cells or immunohistochemical staining of fixed cells by FITC conjugated anti-FLAG antibody (Sigma).

### Immunoprecipitation and Western blot analysis

The 293T cells transfected with various FasL expressing constructs were lysed in 400 μl lysis buffer (50 mM HEPES, pH 7.6; 150 mM NaCl; 1 mM EDTA; 1% NP-40; 1 × protease inhibitor cocktail tablets from Boehringer). For immunoprecipitation of epitope-tagged FasL protein, the cell lysates were incubated with 10 μg/ml mouse-anti-HA antibody (Covance), on ice for 30 minutes. Then, a 20 μl slurry of Protein A Sepharose beads (Pharmacia) was added to each tube and rotated the samples at 4°C for 2 hours. After four washes with the lysis buffer, the beads were resuspended in 30 μl of SDS loading buffer with 0.2 M DTT. The samples were boiled for 5 minutes and the proteins were separated by SDS electrophoresis and transferred to nitrocellulose membranes. These blots were treated with a mouse anti-FLAG antibody (Sigma) followed by an HRP-conjugated secondary antibody. The proteins were detected using the Super signal system (Pierce Chemical Co.).

### FasL-mediated cytotoxicity assay

Fas expressing Jurkat cells cultured in complete RPMI 1640 medium were labeled with PKH2 dye using a PKH2-GL Fluorescent Cell Linker Kit (Sigma). As monitored by flow cytometry, the labeling efficiency was higher than 99%. Aliquots of 0.3 ml of 5 × 10^5^/ml labeled Jurkat cells were dispensed into 24-well plates to serve as target cells. The 293T cells transfected with FasL the day before were harvested and added as effector cells in ratio to target cells of 1:1 or 3:1. To detect dominant interference, 293T cells transfected with 0.2 μg to 0.4 μg of wild type or mutant FasL-expressing plasmid DNAs were used as effector cells at a ratio to target cells of 2:1. The 293T cells transfected with a pCI-neo expression vector with no insert were used as control effector cells. For the cytotoxicity assay involving PBLs from Pt 86 and his father who harbored the same Fas ligand gene mutation, the PBLs were cultured in RPMI medium containing 5 μg/ml PHA-P (Sigma) for 48 hours and used as effector cells. The mixtures of effector and target cells were incubated at 37°C for 3 hours. Then they were harvested, washed once with PBS and analyzed by flow cytometry. A gate was set to enumerate the labeled, live Jurkat cells. The percentage of cell death was calculated as mentioned above in the apoptosis assay.

### Plasma cytokine levels

Cytokine levels were measured in samples of plasma from the Pt. 86, his extended immediate nuclear family members, also in Pt 55C and healthy controls using highly sensitive commercial EIAs kit (R&D Systems) according to the manufacturer's instructions.

## Results

### Clinical histories

Patient 86 (Pt 86) in the NIH ALPS series is a 20-year-old white male with moderate generalized lymphadenopathy and splenomegaly since 18 months of age. He developed thrombocytopenia, neutropenia and anemia at age three, which recurred despite repeated courses of steroids and intravenous immunoglobulin, eventually necessitating splenectomy at age eight. At age ten he developed massively enlarged axillary lymph nodes that were excised. Histological analyses of spleen and lymph node sections revealed a reactive process with numerous follicles and T cell regions that were markedly expanded by infiltrating T cells and non-caseating granulomas. Although mycobacterial infection was suspected, all stains and cultures and TB skin tests were negative and no antimicrobial drugs were dispensed. At age 11 following a respiratory infection he developed granulomatous interstitial pneumonitis that improved spontaneously over several months with no treatment. Elevated liver function tests two- to three-fold above normal were first detected at age 15 years. A liver biopsy showed non-necrotizing granulomatous hepatitis and marked lobular inflammation without fibrosis. His elevated liver function tests have remained stable over time, and a repeat liver biopsy is planned. At age 20 years, he had the onset of what has become chronic recurrent sinusitis that responds to standard antibiotic treatment. His history is remarkable for three episodes of herpes zoster without sequelae at ages 9.6, 12.5 and 15 years. At the time of study he was well, except for continued moderate generalized lymphadenopathy, residual interstitial lung densities and autoimmune hepatitis. He has been followed at the NIH for over 5 years. He has had a history of serum immunoglobulin levels that have been low or borderline low (Table 1), but he has had no serious infections.

**Table 1:** Serum immunoglobulin levels of patient 86

	Age 5			Age 11			Age 20		
			
Igs	Pt86	Norm.	Range	Pt86	Norm.	Range	Pt86	Norm.	Range
IgM	30*	99**	43~196	32	121	52~242	210	156	56~352
IgA	< 7	68	25~154	56	113	45~236	< 10	171	70~312
IgG	450	780	463~1236	544	1007	608~1572	210	994	639~1349

* All values are in mg/dL.

** The normal values are means within 95% confidence limits.

The patient's father had lymphadenopathy as an adolescent and underwent a bone marrow biopsy and other studies that excluded lymphoma. The records of that assessment and tissue samples are unavailable. He has been healthy otherwise except for psoriatic arthritis. The paternal grandparents recall no histories of family members suggestive of ALPS.

An unrelated patient (Pt 55C), is a 13-year-old white female diagnosed with thrombocytopenia with positive anti-platelet antibodies at age 3 years during hospitalization for septic arthritis. She has had several episodes of idiopathic thrombocytopenic purpura treated with intravenous immunoglobulin, but no history of hepatosplenomegaly or chronic adenopathy. Although this patient does not manifest typical ALPS, coincidentally, she has a cousin with ALPS Type Ia. As an immediate patient family member, she is included in our genetic screening.

### Abnormal lymphocyte phenotype in Pt 86

Surface phenotypes of circulating lymphocytes of Pt 86 and members of his family are summarized in Table 2. The patient had a moderate absolute B-cell and T-cell lymphocytosis with increased numbers and percentage of CD4^-^CD8^-^CD3^+ ^(DN) T cells. Notably, most of these were γδ T cells rather than αβTCR^+ ^DNT cells that are characteristic of ALPS [1]. In other Type Ia ALPS patient, accumulation of γδTCR^+ ^DNT has also been reported [28]. Furthermore, CD4^+ ^T cells were elevated which is also not usually the case in ALPS Type Ia. However, we found an increase in HLA-DR^+ ^T cells and a decreased (less than 1) ratio of CD3^+^/CD25^+ ^to CD3^+^/DR^+ ^ratio, which are typical of patients with ALPS [29]. The patient's parents and two siblings showed essentially normal phenotypes, but there were slightly increased B cells in the father and marginal increases in γδ T cells in both unaffected sisters. Similar to the findings in Pt 86, Pt 55C's lymphocyte phenotype also showed a moderate generalized T and B lymphocytosis, with increased numbers of αβ(39, Nl < 18/uL) and γδ (370, Nl < 60/uL) TCR^+ ^CD3^+^CD4^-^CD8^-^T cells,.

**Table 2:** Lymphocyte subpopulations in members of family 86

	**Percent of lymphoid cells **(absolute numbers/mm^3^)					
	
	Pt	Sib1	Sib2	Mother	Father	Controls**
**Total T Cells**						
CD3	64.7(2750)	78.6(1336)	80.3(1767)	71.1(1138)	65.1(1172)	(832–2028)
CD5	62.8(2669)	79.3(1348)	81.8(1800)	72.4(1158)	68.8(1238)	(828–1994)
CD3/ab	51.8(2202)	47.1(801)	75.4(1659)	69.9(1118)	64(1152)	(620–2010)
CD3/gd	12(510)	5.3(90)	5.3(117)	0.9(14)	0.9 (16)	(9–166)
**T Cell Subsets**						
CD4	36.1(1534)	43.3(736)	52(1144)	41.9(670)	40.6(731)	(480–1339)
CD8	26.7(1135)	31.8(541)	31.6(695)	33.5(536)	29.6(533)	(351–911)
CD3^+^CD4^-^CD8^-^	9.7(412)	4.6(78)	4.9(108)	1.1(18)	1.3(23)	(9–122)
DNT/ab*	1.1(47)	0.8(14)	1.1(24)	0.5(8)	0.5(9)	(2–18)
DNT/gd	7.9(336)	3.9(66)	3.4(75)	0.6(10)	0.7(13)	(5–59)
**T Cell Activation**						
CD4^+^/HLA-DR^+^	14.2(604)	1.6(27)	2.3(51)	2.8(45)	2.7(49)	(< = 95)
CD4^+^/CD25^+^	5.2(221)	16.1(274)	11(242)	19.7(315)	22.1(398)	
**Total B cells**						
CD20	18.9(803)	8.8(150)	8.1(178)	5.9(94)	19.6(353)	(88–330)

*DNT: Double-negative CD4^- ^CD8^- ^T cells, either TCRαβ^+ ^or TCRγδ^+^

** 95% confidence intervals for adult normals.

### Cytokine profile in Pt 86

ALPS Type Ia is associated typically with significant elevations of serum and lymphocyte IL-10, and relatively low or normal levels of Th1 cytokines [30]. To help determine whether Pt 86, his family members, or Pt 55C had these features of ALPS, their sera were analyzed with sensitive immunoassays during the years of our clinical follow up examination. The median level of IL-10 in normal individuals in our laboratory is 3.4 pg/ml [30]. We observed moderately elevated levels of IL-10 in Pt 86 (mean = 20 pg/ml, 14~27 pg/ml over 5 years), in his father (mean = 9.5 pg/ml, 5~14 pg/ml over 2 years), but levels were normal in Pt 86's extended-nuclear family including the FasL-mutant carrier grandmother, as well as in Pt 55C. Circulating IL-2 level in Pt 86 was slightly elevated (mean = 28 pg/ml, 11~79 pg/ml over 5 years), 0~11 pg/ml (during 2 years) in his father, and undetectable in his mother, and 41 pg/ml in Pt 55C (in the range in unaffected ALPS family members). In summary, Pt 86 shows a cytokine profile that is less clearly oriented towards a Th2 response than that is typical in ALPS Type Ia patients [31].

### Identification of FasL mutations

**Figure 1:**
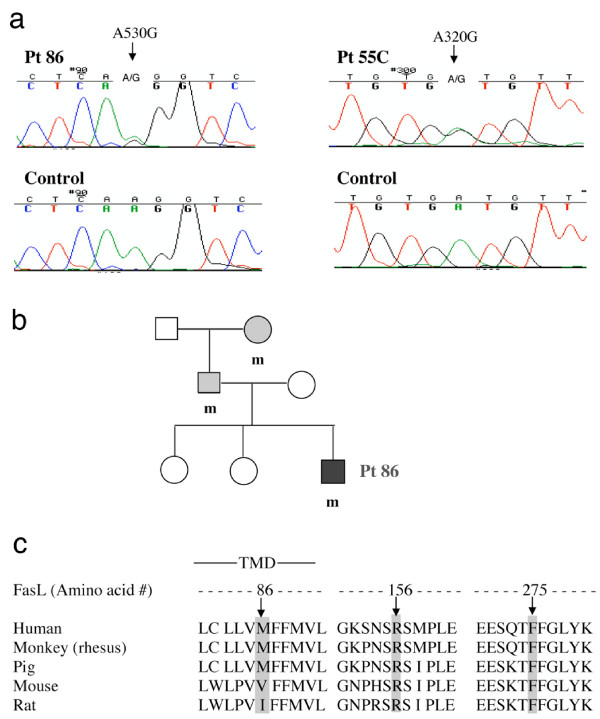
Fas ligand mutations in the Pt 86, Pt 55C and the *gld *mouse. Sequencing of genomic DNA shows a heterozygous A530G mutation in Pt 86 and a A320G mutation in Pt55C depicted in panel **(a) **for double peaks of A and G from normal and mutant alleles amplified from genomic DNA. The family pedigree in panel **(b) **reveals the same mutation was found in his father, and paternal grandmother (labeled with m) but not in his healthy mother or two sisters. Black, the affected individual; gray, individuals with the mutation but not the full picture of ALPS. This mutation leads to an amino acid change of A156G, the heterozygous A320G mutation found in Pt 55C FasL genomic DNA leads to an amino acid change M86V in the transmembrane domain (TMD), and the *gld *mutation in mouse is shown at amino acid site 275, all compared to the wild type sequences at these sites in 5 species, as aligned in panel **c**.

Given the generally typical history, clinical features, and immunophenotype in Pt 86, the diagnosis of ALPS was expected. Yet, DNA sequencing revealed no mutations in the genes encoding Fas or caspase-10, which have been previously identified to cause ALPS [3,4,8]. We therefore, sought mutations in other apoptosis genes in these individuals and in 38 others in whom a genetic basis for ALPS had not been defined. The only mutations found in *TNFSF6 *to date were in these two subjects. Pt 86, his father and paternal grandmother share a heterozygous FasL A530G mutation (Figure 1a,b) that leads to substitution of arginine to glycine at peptide 156 (R156G). The mutation was not observed in the patient's healthy mother and two sisters (Figure 1b). The arginine residue in FasL is conserved among mouse, rat, pig, monkey, and humans (Figure 1c) suggesting that it may be important for FasL function.

Pt 55C had no Fas or caspase-10 abnormalities, but carried a heterozygous A320G base change (Figure 1a) that causes a methionine to valine substitution at amino acid 86 (M86V) in the FasL transmembrane domain (Figure 1c) [6]. This residue is normally a valine in the mouse and an isoleucine in the rat, suggesting that any hydrophobic, or weakly polar residue is acceptable. Thus, we hypothesize that the change in Pt 55C is not relevant to FasL function. Neither of the base changes found in Pt 86 or Pt 55C was observed in the FasL sequences of 100 chromosomes from 50 normal individuals.

We chose to compare these human sequence alterations to the *gld *mouse that carries a homozygous mutation replacing phenylalanine with leucine at position 273 (equivalent to F275L in human FasL), which is located 7 amino acids upstream of the stop codon [7]. This residue is absolutely conserved within a region of FasL that is highly homologous among at least 5 species (Figure 1c) and is critical for binding of FasL to its Fas receptor [32].

### Molecular modeling of the FasL mutations

**Figure 2:**
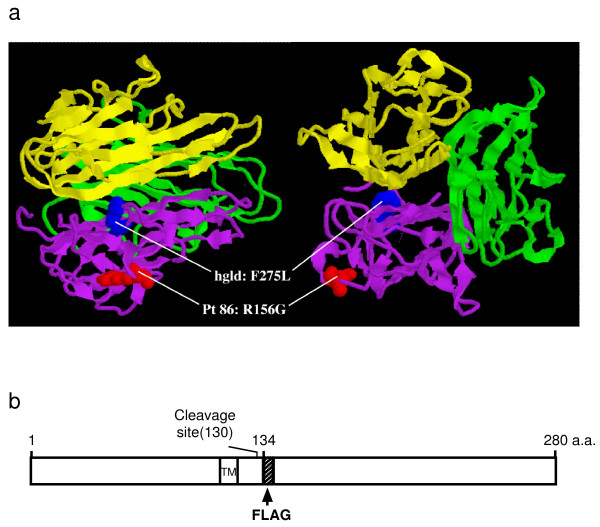
**(a) **Molecular modeling of the extracellular, soluble portion of rat FasL. The soluble rat FasL trimer fragment was visualized based on the structure of TNF-α [33]. The Pt 86 FasL mutation is labeled in red and the h*gld *mutation is labeled in blue in side (left) and front (right) projections. The Pt 55C FasL mutation is not indicated in this model since it is located in the transmembrane domain, which is outside of the region in the model depicted. **(b) **A diagram of cloning strategy for epitope-tagging Fas-ligand protein.

To help understand the biological implications of the mutations found in Pt 86 and Pt 55C, we compared human FasL sequences with those of other species, as well as the *gld *mice. Alignment of FasL amino acid sequences showed 74% homology among human, mouse, and rat [6]. We also positioned R156 on the molecular model of soluble rat FasL fragment trimer and a Fas/FasL interaction model based on the 3-dimensional X-ray crystallographic structure of tumor necrosis factor alpha (TNF-α) [33,34]. We found that the Pt 86 substitution is located in a surface-exposed area in the extracellular domain near the predicted Fas/FasL interface (labeled red in Figure 2a). The mouse *gld *substitution at the homologous site in human FasL (h*gld*, Phe275Leu; labeled in blue in Figure 2) is predicted by molecular modeling to lie on another loop of the protein in proximity to the FasL trimer interface. This model predicts that the mutation in Pt 86 may directly affect Fas-FasL interactions and impair apoptotic signaling. The heterozygous A320G mutation in Pt 55C, however, leads to a conservative substitution of Met to Val at amino acid 86 in the predicted transmembrane domain (TMD), a region not included in this molecular model. Given the location, the conservative nature of the mutation in Pt 55C, and the fact that valine normally occupies this position in mice, this mutation should have no impact on apoptosis.

### Fas-mediated apoptosis is normal in patients with FasL mutations

**Figure 3:**
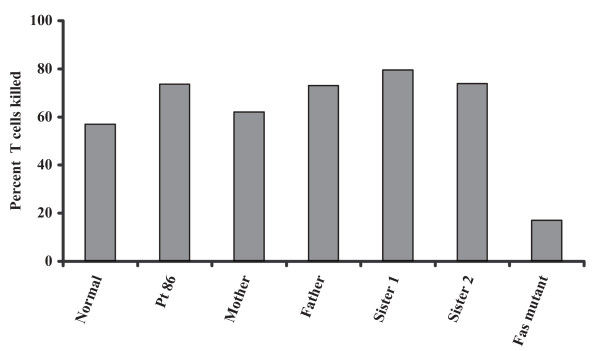
Fas-mediated T-cell apoptosis in Family 86. The percentage of T cells killed by the treatment with an agonistic anti-Fas antibody is shown for the patient, members of his extended family, a normal control, and an ALPS Type Ia patient with a mutation in the Fas death domain. Percent cell loss is calculated as (1-number of cells with antibody stimulation/number of cells without stimulation) 100%. One representative of three repeated experiments is shown.

Activated PBLs from Pt 86 and his nuclear family, and from Pt 55C, were cultured in the presence of IL-2 and stimulated to undergo apoptosis with an agonistic anti-Fas antibody. The level of T cell loss for Pt 86 and his relatives were similar to the normal subject (Figure 3). As a control, defective apoptosis was clearly observed using lymphocytes from an ALPS Type Ia patient. Pt 55C PBLs also showed no defect in Fas-induced killing (not shown). Normal Fas-mediated apoptosis induced by direct receptor stimulation would be expected to be unaffected in persons harboring FasL mutations but normal Fas. Thus, there were no apparent apoptotic defects in downstream Fas signaling. It was then critical to directly analyze the ability of FasL from these individuals to trigger apoptosis.

### Impaired cytotoxicity of mutant FasL from Pt 86

**Figure 4:**
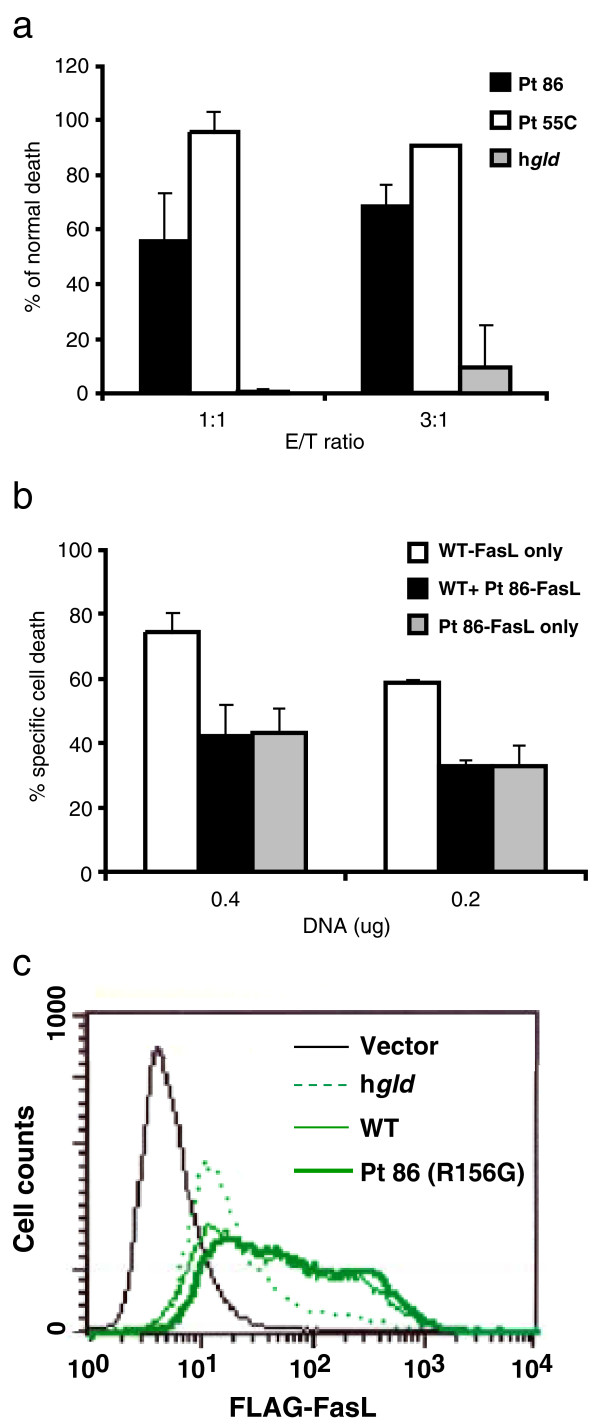
Cytotoxic effects of transfected FasL constructs on Jurkat cells. Cytotoxicity is reflected by the percentage of Fas-positive Jurkat cells killed by co-culture with transfected 293T cells. **(a) **Ratios of 1:1 or 3:1 were used for 293T cells (effectors) transfected with either of 4 constructs (WT; Pt 86, black; Pt 55C, open; and h*gld*, shaded) to Jurkat cells (targets). Averages of % cell death and error bars are shown as normalized to that of WT FasL-induced death. Data represent at least 3 experiments. **(b) **Cotransfection of the Pt 86 (R156G) mutant FasL with wild type FasL. 293T cells were transfected with a total of 0.4 or 0.2 μg of wild-type FasL only (open bars); with Pt 86 mutant mixed with wild-type FasL at ratio of 1:1 (black bars); and with Pt 86 mutant FasL only (shaded bars). The ratio of effector to target cells was 2:1 in this experiment. Means and error bars of the percentage Jurkat cells death are shown. Data represent two independent duplicate experiments. **(c) **Surface staining of FLAG-tagged FasL in 293T cells transfected with empty vector, h*gld-, WT*-, and Pt 86- FasL as indicated.

FasL-mediated apoptosis was evaluated by determining the ability of effector cells transfected with various FasL expression vectors to kill Fas-bearing Jurkat cells. FLAG-tagged FasL expression constructs were transfected into 293T cells and tested as effectors. As shown in Figure 4a, at effector to target cell ratios of 1:1 or 3:1, the construct expressing Pt 86 mutant FasL was substantially less effective at killing Jurkat cells as compared with the wild type. By contrast, the Pt 55C construct induced normal levels of apoptosis. The human FasL bearing the murine *gld *mutation (h*gld*) exhibited essentially no cytotoxic activity. The transfection efficiency and protein expression from all 4 FasL constructs were similar by Western blot analysis and immunohistochemical staining of fixed cell lysates (data not shown).

Interestingly, FasL surface expression by flow cytometry using the h*gld *construct was substantially less than the other three constructs (Figure 4c). Thus, the lack of killing of Jurkat cells by the h*gld *FasL construct may due, at least in part, to low levels of FasL expression on the cell surface.

### Dominant interference of the mutant FasL from Pt 86

Previous studies indicated that most Type Ia ALPS patients carry heterozygous Fas mutations, and that the mutated Fas molecules have dominant interfering effects on the ability of normal Fas molecules to initiate apoptosis [4]. To address the mechanism by which the heterozygous FasL mutation in Pt 86 leads to defective apoptosis and ALPS, we examined the possibility that abnormal FasL interferes dominantly with normal FasL. We then co-transfected Pt 86 and wild type FasL constructs and observed that the cytotoxicity was indeed much lower than that induced by the wild type FasL construct alone (Figure 4b). In fact, an approximately equimolar mix of the wild type and mutant plasmids yielded no greater cell loss than mutant FasL alone (Figure 4b). Constructs expressing h*gld *or Pt 55C variants of FasL had no effect on killing by wild-type FasL (data not shown). Thus, Pt 86 mutant FasL interferes in a dominant fashion with killing from normal FasL.

### PBLs from Pt 86 and his father have diminished cytotoxic activity

**Figure 5:**
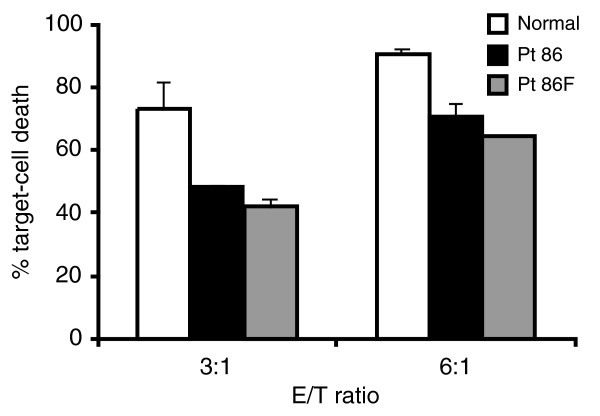
Cytotoxic effects of patient PBLs on Jurkat cells. The activated PBLs from normal controls (open), Pt 86 (FasL R156G, black), and father of Pt 86 (shaded) were used as the effector cells to mix with Jurkat (target) cells at the E/T ratios shown. Means and error bars of % target cell death are shown, data represent two independent experiments.

Having shown that FasL of Pt 86 is impaired in initiating apoptosis, it was important to show that PBLs taken directly from this patient are similarly defective in inducing Fas-mediated cell death. We therefore assessed whether PBLs from Pt 86, his father and normal individuals could induce the death of Fas-expressing Jurkat cells. Cell death was reproducibly reduced for both Pt 86 and his father, compared to normal cells (Figure 5). It is therefore clear that action of the wild-type FasL allele in the patient and his father is impaired by the presence of the mutant allele.

### Mutant FasL interacts with wild type FasL

**Figure 6:**
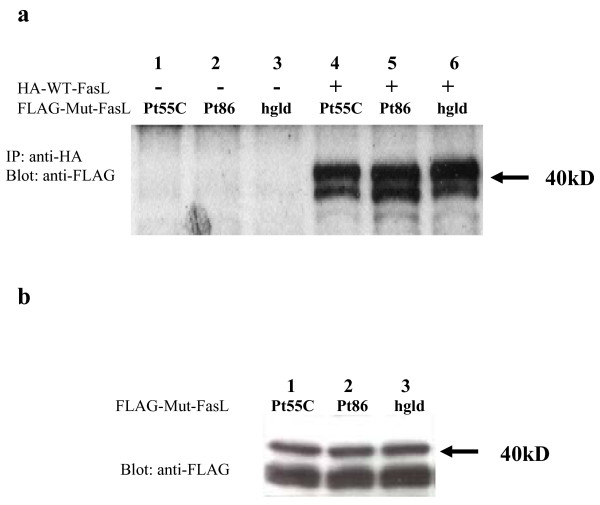
Physical association of FLAG-tagged mutant FasL with HA-tagged wild type FasL. **(a) **Lanes 1, 2 and 3 contain extracts from cells transfected with the FLAG-tagged mutant FasL constructs (A320G, A530G and h*gld*) alone and lanes 4, 5 and 6 are from cells co-transfected with one of FLAG-tagged mutant FasL constructs and the HA-tagged wild type FasL construct. An anti-HA antibody was used for the immunoprecipitation and an anti-FLAG antibody was used for Western blot analysis. The double bands appearing at about 40 kD represent two FasL isoforms with different glycosylation patterns. **(b) **Western blot analysis of transfected cell lysates. The FLAG-tagged FasL with the mutations (lanes 1–3) were expressed in 293T cells, and FasL expression was detected by Western blotting with an anti-FLAG antibody.

Fas mediates lymphocyte apoptosis as a pre-associated homotrimer [14]. Evidence indicates that FasL spontaneously assumes a homotrimeric structure to symmetrically engage the trimeric Fas assembly [34]. It may, therefore, be possible to detect the physical association of mutant and/or wild type FasL proteins into an oligomeric structure which could explain the dominant inhibition of apoptosis in people with heterozygous FasL mutations. We expressed in 293T cells of various forms of FasL with HA or FLAG tags inserted right after the metalloprotease cleavage site of FasL protein (Figure 2b). We found that all three FLAG-tagged FasL mutant proteins associate with the HA-tagged wild type FasL in immunoprecipitation experiments (Figure 6). Control western blots on cell lysates using anti-FLAG antibody showed that the FasL with mutations in Pt 86, Pt 55C or h*gld *were expressed comparably to that of *WT *FasL (Figure 6b). We observed no cross-reaction of anti-FLAG and anti-HA antibodies (data not shown). Thus the Pt 86 and h*gld *FasL proteins have rather normal overall conformations and can associate with *WT *FasL. The dominant interfering effect we observe with the Pt 86 heterozygous FasL mutation implies that the amino acid substitutions corrupt receptor interaction and apoptotic signaling, but still permit oligomerization of the mutant FasL chain with the wild-type chain. Hence, the mutant FasL chains prevent normal FasL chains from functioning and therefore dominantly interfere with apoptosis.

## Discussion

Here we report a young adult, Pt 86, with ALPS due to a point mutation in the *TNFSF6 *gene encoding FasL. He meets the defining criteria for ALPS by exhibiting chronic lymphadenopathy, splenomegaly, autoimmune hepatitis, elevated numbers of CD4^-^CD8^-^TCRαβ^+ ^T cells and an apoptosis defect. However, unlike most patients with ALPS, he has no abnormality of the Fas receptor gene *TNFRSF6*, or of downstream intracellular signaling molecules that transmit the death signal. Rather, his FasL is defective. Thus, he can be classified as having a previously undocumented form of heterozygous ALPS (Type Ib) reserved by analogy with *gld *mice for cases arising because of FasL mutations.

Pt 86 has notable features that are not typical of ALPS patients with Fas mutations. These include hypogammaglobulinemia and granulomatous infiltration of the spleen, lymph nodes and liver. His hypogammaglobulinemia in this setting may be due to an abnormality in T cell help rather than a primary B cell defect. Paradoxically both the patient and his father, who carries the same FasL mutation on one of his alleles, have elevated circulating B cell numbers. In addition, Pt 86, but not his father, has a decreased percentage of CD3^+^CD25^+ ^T cells, known to represent a regulatory subset, while the percentage of his CD3^+ ^HLA-DR^+ ^T cells is elevated, consistent with ongoing activation in vivo. Clinical evidence for Pt 86 in support of a cellular immune defect is his history of difficulty in controlling multiple viral infections: he has suffered from cutaneous warts and chronic lesions of molluscum contagiosum; and he had three discrete episodes of herpes zoster in the absence of immunosuppressive medications. Recent studies suggest that FasL ligation to Fas on T cells can act as a positive costimulatory signal in antigen-specific T cell activation [36,37]. Moreover, direct Fas engagement has also been observed to provide a costimulatory effect on T cell proliferation [38]. Thus, ineffectual Fas-FasL interactions might produce a T cell activation defect in a fashion similar to that seen with mutations in CD40-ligand (CD154) in X-linked hyper-IgM syndrome. Interestingly, the adult immunoglobulin levels for Pt 86 suggest a partial isotype switch defect with low IgA and IgG and IgM in the upper range of normal. However, if this were of substantial magnitude, one should observe T cell activation defects in patients with heterozygous and homozygous Fas mutations, nevertheless, they are substantially milder than those in CD154 deficiency. We have seen instances of herpes zoster infection and poor antibody responses following immunizations in ALPS Type Ia patients. However, cellular immune defects are not characteristic of *gld *mice indicating that there is a partial signaling effect or some other reason for these clinical findings [7].

Pt 86 originally presented with prolonged granulomatous interstitial pneumonitis that developed after a respiratory infection, and granulomas were evident on histological evaluation of his splenic, lymph node and liver tissues. Interestingly, after some viral infections the FasL-deficient *gld *mice also develop inflammatory responses in the pulmonary interstitium that persist long after the infectious agent has disappeared [39]. Nonetheless, the cause of the granulomas in Pt 86 remains unclear. No infectious etiology was found and the condition remained stable without antimicrobial intervention. Although his histology resembled sarcoidosis, Pt 86 lacked the cutaneous, neurological, and ocular features of that disease. Further patients with ALPS Type Ib must be studied in order to elucidate the relationship between FasL defects and granulomas. Lymph node biopsy of the FasL defective patient described by Del-Rey *et al *[19] revealed sinus histiocytosis but no granulomas. Since their patient had a complete loss of function while Pt 86 has only partial loss of FasL function, it is possible that the difference in histology reflects this qualitatitive difference in function.

Another interesting difference between the patient reported by Del-Rey *et al*. [19] and patient 86 lies in the impressive history of recurrent infections in the former despite chronically elevated immunoglobulins. In contrast, Pt 86 has suffered from relatively few infections despite his pan-hypogammaglobulinemia. This difference may reflect virtually absent FasL function in the Del-Rey patient and partial function in Patient 86. However, like the Del-Rey patient, Pt 86 has experienced recurrent episodes of herpes zoster suggesting that this patient has altered cellular and humoral immune function as suggested by his depressed immunoglobulin levels.

The identification of Pt 86 and the present studies of the basis and mechanisms by which his mutation impairs apoptosis afford new insights into FasL biology. While the *lpr *and *gld *murine mutations in Fas or FasL, respectively manifest their phenotype in the homozygous state [7,40], most ALPS patients including Pt 86 carry heterozygous mutations [3,4]. Because human populations are usually not inbred, it was important to understand how heterozygous mutations impair cell death effector functions. Since Fas transduces the death signal as a homotrimeric complex, we showed that individual mutant Fas proteins, as seen in nearly all patients with ALPS Type Ia, dominantly inhibit apoptosis mediated by wild type Fas [4]. Now corresponding analyses of mutations in FasL have been made possible by the discovery of Pt 86.

Molecular modeling predicts that this A530G mutation is at a surface-exposed residue near the Fas/FasL interface, suggesting that the mutation directly impairs Fas/FasL interactions. We showed that the mutant FasL chain associates readily with the wild-type chain and thereby dominantly interferes with apoptosis induction by wild-type FasL. By contrast, the *gld *mutation likely impairs Fas binding indirectly through disrupting the structure of the FasL trimer. The mutation in Pt 55C is a highly conservative one in which the methionine normally found at position 86 within the transmembrane domain of FasL in humans is replaced with another hydrophobic residue, valine, which is in fact found at this site in mice. Thus, it was not surprising that the FasL of Pt 55C can associate successfully with wild type FasL and does not interfere with the apoptotic killing mediated by wild type FasL.

Assuming that the wild type and Pt 86 mutant forms of FasL protein are synthesized equally, have equal stability (which would be difficult to confirm experimentally), and participate in trimer formation at random, the trimeric complexes generated by combinations of these molecules would include one-eighth of them comprised entirely of mutant FasL peptides, three quarters with one or two mutant FasL peptides, and one-eighth with only wild type FasL. Thus, some residual amount of normal FasL trimers would be expected and this might explain the residual apoptosis in PBLs of Pt 86 and his father (Figure 5).

The father of patient 86 carries the same mutation as his son, and while he is reported to have had significant adenopathy during childhood, he does not currently manifest sufficient clinical features for the diagnosis of ALPS to be made. Variable expressivity of heterozygous mutations was confirmed early in our studies of ALPS Type Ia families with Fas mutations [4]. The present data reveal parallel findings in ALPS Type Ib families with FasL mutations. Further investigations into other gene contributions may reveal the molecular basis for variable penetrance in ALPS.

## Conclusion

This report shows that an amino acid substitution in FasL generates a mutant protein that binds to and dominantly interferes with the function of the normal FasL thus explaining how a heterozygous FasL gene mutation causes ALPS Type Ib.

## Abbreviations

ALPS – Autoimmune Lymphoproliferative Syndrome

TNF – tumor necrosis factor

FasL – Fas ligand

kD – kiloDalton

PCR – polymerase chain reaction

TB – tuberculosis

Ig – immunoglobulin

Nl – normal

DNT – double negative T cells

HLA – human leukocyte antigen

## Competing interests

The author(s) declare that they have no competing interests.

## Authors' contributions

LLB and GP performed the molecular and biochemical studies.

LZ participated in the design and carry out of molecular, biochemical and transfection experiments, assisted in the interpretation of the data, and the revision of the manuscript.

JKD, JMP, and SES carried out clinical studies and participated in the interpretation of the data and writing of the manuscript.

RMS assisted in the structural analysis of the mutations.

CM, VR, and JMD selected and evaluated patients and carried out the initial characterization of the defect.

TPA participated in the design of the study and helped to draft the manuscript.

MJL participated in the design of experiments, interpretation of the data, and the preparation and submission of the manuscript. All authors read and approved the final manuscript.

All authors approve the final manuscript.

## Pre-publication history

The pre-publication history for this paper can be accessed here:


